# Association of microRNA biosynthesis genes XPO5 and RAN polymorphisms with cancer susceptibility: Bayesian hierarchical meta-analysis

**DOI:** 10.7150/jca.37150

**Published:** 2020-02-03

**Authors:** Yi Shao, Yi Shen, Lei Zhao, Xudong Guo, Chen Niu, Fen Liu

**Affiliations:** 1 Department of Epidemiology and Health Statistics, School of Public Health, Beijing Municipal Key Laboratory of Clinical Epidemiology, Capital Medical University, Beijing, China.; 2 Department of Molecular Physiology and Biophysics, Holden Comprehensive Cancer Center, University of Iowa Carver College of Medicine, Iowa City, IA, United States.

**Keywords:** Bayesian hierarchical meta-analysis, XPO5, RAN, polymorphism, cancer

## Abstract

XPO5/RAN-GTP complex mediates the nuclear transport of pre-miRNAs in the miRNA processing system, its altered expression is indicated to be correlated with cancer risk. Several studies have inspected the association between XPO5 or RAN polymorphisms and the risk of various cancers, but the findings remain controversial. A Bayesian hierarchical meta-analysis was carried out to review and analyze the effect of XPO5 and RAN polymorphisms on cancer risk. The association was estimated by calculating the logarithm of odds ratio (Log OR) and 95% credible interval (95% CrI). The expression quantitative trait loci (eQTL) analysis was used for *in silico* functional validation of the identified significant susceptibility loci. Consequently, 38 case-control studies (from 27 citations) with 27,459 cancer cases and 25,151controls were included in the meta-analysis of the five most prevalent SNPs (rs11077 A/C, rs2257082 G/A, rs3803012 A/G, rs14035 C/T, rs3809142 C/T). In the XPO5 gene rs11077 SNP, the minor C allele significantly increased the risk of cancer (Log OR = 0.120, 95% CrI = 0.013, 0.241), and a strong association between rs11077 SNP and cancer risk was also found in the dominant model (CC + AC vs. AA: Log OR = 0.132, 95% CrI = 0.009, 0.275). In addition, the minor GG genotype allele of the RAN gene rs3803012 SNP significantly increased the cancer risk (Log OR = 0.707, 95% CrI = 0.059, 1.385). Statistically significant associations between rs3803012 SNP and cancer risk were also observed in the recessive model (GG vs. AG + AA: Log OR = 0.708, 95% CrI = 0.059, 1.359). Furthermore, the eQTL analysis revealed that rs11077 SNP was significantly correlated with XPO5 mRNA expression, which provided additional biological basis for the observed positive association. Our results suggest that XPO5 rs11077 may be a possible functional susceptibility locus for cancer risk.

## Introduction

MicroRNAs (miRNAs) are a highly conserved class of small, noncoding RNAs, which mediate post-transcriptional gene silencing 1. Over the past decade, they have increasingly been recognized to be involved in the initiation and progression of human carcinogenesis 2. The biosynthesis of miRNAs involves a multiple-step process that starts in the nucleus of the cell, where miRNA genes are initially transcribed as primary miRNAs (pri-miRNAs), and then converted into precursor miRNAs (pre-miRNAs). Secondly, with the assistance of GTP-binding nuclear protein Ran/exportin-5 (XPO5) complex, the pre-miRNAs are exported from the nucleus to the cytoplasm, where the mature miRNA molecule exerts its main function 3, 4.

During the miRNA maturing processing, the XPO5/RAN-GTP complex mediates the nuclear transport of pre-miRNAs, which are crucial components. XPO5, a member of the nucleo-cytoplasmic exportins, is related to the human export receptor that uses the Ran-GTPase to control cargo association 5, 6. Studies have shown that the overexpression of XPO5 is found to improve transport efficiency and further enhance miRNA activity, while the downregulation of XPO5 leads to a loss of pre-miRNA function 7, 8. RAN encodes a small G protein that is crucial for the translocation of RNA and proteins through the nuclear pore complex. If the RAN is depleted, the output of the pre-miRNA will be greatly reduced 9, 10. Therefore, impaired miRNA processing caused by the dysregulation expression of miRNA biosynthesis genes XPO5 or RAN can noticeably promote the tumorigenesis 11.

Increasing evidence proposed shows that single-nucleotide polymorphisms (SNPs) in core components of miRNA biogenesis may impair or enhance miRNA processing efficiency or function, which can function as an oncogene or tumor suppressor 12. Formerly, several studies have been conducted to assess the association between XPO5 and RAN SNPs and cancer susceptibility in diverse populations. However, the conclusions of the findings remain inconsistent. Hence, a meta-analysis is required to combine data from all the individual studies to obtain a more comprehensive and effective estimation. Previous studies have reviewed the relationship between polymorphisms of miRNA processing genes and cancer risk through classical meta-analysis approach 13. However, the results did not indicate a correlation between SNPs in XPO5 and RAN genes with cancer risk. This might be as a result of the small number of articles included. Indeed, classical meta-analysis requires the initial sample to be large enough to ensure the asymptotic normality of the effect size and, to further obtain accurate and realistic results 14. Bayesian hierarchical meta-analysis, however, provides a more accurate pooled effect size compared to classical meta-analysis approaches, especially in situations with a small number of studies 15. Therefore, in the present study, we carried out a Bayesian hierarchical meta-analysis including newly published articles to find a vivid and precise association between SNPs in XPO5 and RAN genes with cancer risk based on all available eligible studies. We also used expression quantitative trait loci analysis (eQTL) to validate the potential function of the identified significant susceptibility loci.

## Materials and methods

### Retrieval strategy

To identify all potentially eligible publications, PubMed, PubMed Central (PMC), Web of science, Embase, China National Knowledge Infrastructure (CNKI), Chinese Wanfang databases, Wiley, Google Scholar, Cochrane, the Cochrane Central Register of Controlled Trials were searched, using a combination of the following keywords: 'XPO5/ exportin 5/ exp5/ RAN/ ARA24/ Gsp1/ TC4'; 'SNP/ polymorphism/ variation/ variant'; and 'tumor/ cancer/ carcinoma/ neoplasm'. The search was limited to articles published in English or Chinese through April 9, 2019. References of the relevant literature and review articles were also evaluated to identify all potentially eligible articles. This meta-analysis was carried out in accordance with the Preferred Reporting Items for Systematic Reviews and Meta-Analyses (PRISMA) statement (Supplementary PRISMA 2009 Checklist) 16.

### Inclusion and exclusion criteria

Eligible publications were selected based on the following inclusion criteria: (i) evaluation of genetic association between XPO5 or RAN and susceptibility to cancer; (ii) a case-control designed study; Articles meeting the following criteria were excluded: (i) reviews, meta-analyses, conference reports, or editorial articles; (ii) duplicate records; (iii) no available data to extract; (iv) the control subjects exhibited a departure from Hardy-Weinberg equilibrium (HWE).

### Data extraction and Quality assessment

The following information was extracted by two reviewers independently and disagreement was solved through discussion: first author's name, publication year, country, ethnicity, cancer type, polymorphisms, sample size of cases and controls, genotype distribution, source of control groups (population-based (PB) or hospital-based (HB)), genotyping method, HWE in controls. If more than one type of cancer or multistage research was involved in a single article, data for each type of cancer was extracted independently. When the data in eligible articles was unavailable, we tried our best to contact the corresponding authors for original data. Quality assessment of articles was conducted using the Newcastle-Ottawa Quality Assessment Scale (NOS) 17. NOS scores range from 0 to 9. To the best of our knowledge, there is no established cut-offs for low, moderate and high quality. Hence, we have relied on previous literature 18 to define low quality as a score ≤ 5, moderate quality as a score between 6 and 7, and high quality as a score between 8 and 9.

### Statistical methods

In this meta-analysis, the following comparisons for XPO5 and RAN polymorphisms were evaluated in five common genetic models including allele model (V vs. W) (W for wild allele, V for variation allele), heterozygote model (WV vs. WW), homozygote model (VV vs. WW), dominant model (WV+VV vs. WW), and recessive model (VV vs. WW+WV).

### Bayesian meta-analysis method

The Bayesian meta-analysis is a Bayesian modeling method which determines the prior distribution with hierarchical prior distribution, and then does the statistical inference 19. Compared with the classical meta-analysis, the Bayesian hierarchical random-effect model can obtain accurate pooling effects, especially in situations with a small number of studies 14, 20-22. A Bayesian approach allows one to coherently process the uncertainty in the heterogeneity parameter while focusing on inference for the effect parameters, and interprets the results more intuitively 23. In order to compare the effect magnitude between different studies, the pooled logarithmic odds ratio (Log OR), between-study standard deviation (*τ^2^*) and their respective 95% credible intervals (CrIs) are estimated. The 95% CrI is the Bayesian equivalent for standard confidence intervals. In particular, the model supposes that the mean of the Log ORs has a low-informative normal distribution (mean = 0, variance = 100) and the variance of the Log ORs has a low-informative inverse-gamma distribution (0.01, 0.01) 24. Sensitivity analyses with different choices of low-information prior distributions showed the robustness of this choice 20. In addition, we estimated the* I*^2^ statistic, which is used to measure the total variation 25. Forest plots, which illustrate Log ORs and 95% CrIs for both the individual trials and the pooled results, were included in our meta-analysis. Moreover, the heterogeneity plot displayed the joint posterior density of the two parameters, Log OR and *τ* parameters, with darker shading corresponding to higher probability density. All the statistical analyses were calculated using “bayesmeta” R package (https://cran.r-project.org/web/packages/bayesmeta/index.html).

### In silico functional validation

To validate the potential impact of the cancer risk SNP, we examined its association with the expression of corresponding genes using eQTL databases. The eQTL analysis was performed by using the genotyping and expression data of lymphoblastoid cells from 373 European individuals available in the 1000 Genomes Project 26. Considering that many eQTLs are population-specific, we also extracted eQTL data of East Asian individuals from a study by Stranger et al. 27, in which genome-wide mRNA expression in lymphoblastoid cell lines of 726 individuals from eight global populations in the HapMap3 project was analyzed. Seventeen cases of hepatocellular carcinoma genotype and gene expression data were obtained from the Gene Expression Omnibus (GEO) database (https://www.ncbi.nlm.nih.gov/geo) (GSE65373). Choosing hepatocellular carcinoma and breast cancer as representatives, we also downloaded mRNA sequencing datasets of 154 paired cancer tissue samples and normal adjacent tissue samples from The Cancer Genome Atlas (TCGA-LIHC and TCGA-BRCA) (https://tcga-data.nci.nih.gov/tcga/). A linear regression model was performed to evaluate the correlation between SNPs and specific mRNA expression levels. A paired t-test was used to test for the differences in gene mRNA expression levels between cancer tissue and adjacent normal tissue from the TCGA database. All analyses were performed using R (version 3.5.1).

## Results

### Study characteristics

**Figure 1:**
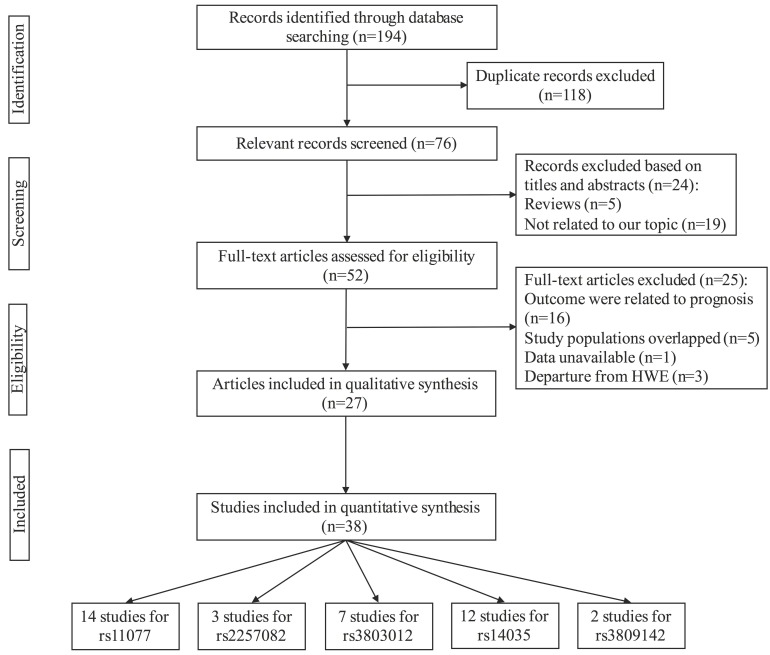
The flow chart of identification for studies included in the meta-analysis

The process of selecting eligible studies is depicted in Figure 1. A total of 194 articles were identified based on our search strategy, 118 of the articles were duplicates. After a screening of the titles and abstracts, 24 articles were excluded for irrelevant information (5 were reviews, 19 were not related to our topic). We eliminated 25 records after browsing the full text of the remaining 52 articles (16 were related to prognosis; 5 had overlapping study populations; 1 for unavailable data; 3 were departure from HWE). Finally, 38 studies from 27 articles with 27,459 cases and 25,151 controls were included in our meta-analysis 28-54. Table 1 shows the characteristics and relevant data of the included studies. The detail of NOS scores for every included study was shown in Table S1. In summary, 38 eligible case-control studies, five SNPs of XPO5 or RAN genes were investigated in the eventual analysis. In XPO5, the analyzed SNPs were rs11077 A/C, rs2257082 G/A; while in RAN, the analyzed SNPs were rs3803012 A/G, rs14035 C/T, rs3809142 C/T.

### Quantitative synthesis

The main results of Bayesian hierarchical meta-analysis were calculated as the median of the marginal posterior distribution of the Log ORs and *τ* parameters. On the basis of the Bayesian hierarchical meta-analysis, XPO5 rs11077 and RAN rs3803012 SNPs were significantly associated with the risk of cancer (Table 2).

**Figure 2:**
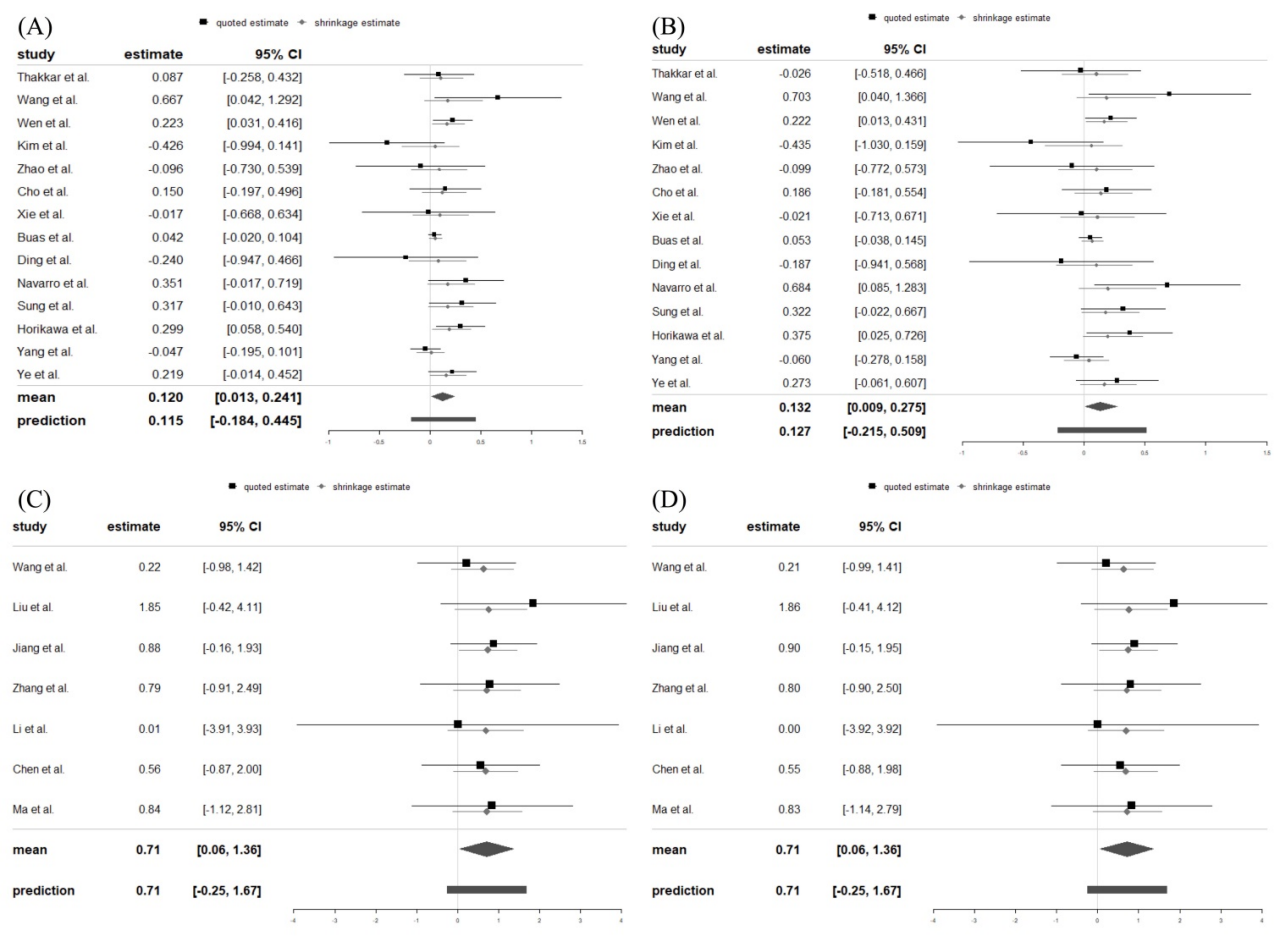
Forest plots displayed Log ORs and 95% credible intervals for both the individual trials and the pooled results. (A) rs11077: C vs. A; (B) rs11077: CC+AC vs. AA; (C) rs3803012: GG vs. AA; (D) rs3803012: GG vs. AG+AA. OR: odds ratio

In the XPO5 gene rs11077 SNP (Figure 2A), the minor C allele significantly increased the risk of cancer (Log OR = 0.120, 95% CrI = 0.013, 0.241). A strong association of rs11077 SNP with cancer risk was also found in the dominant model (CC + AC vs. AA: Log OR = 0.132, 95% CrI = 0.009, 0.275) (Figure 2B). In addition, the minor GG genotype allele of the RAN gene rs3803012 SNP (Figure 2C) significantly increased the cancer risk (Log OR = 0.707, 95% CrI = 0.059, 1.385). Statistically significant associations between rs3803012 A/G SNP and cancer risk were also observed in the recessive model (GG vs. AG + AA: Log OR = 0.708, 95% CrI = 0.059, 1.359) (Figure 2D). However, alleles and genotypes in other polymorphisms of XPO5 and RAN genes were not significantly associated with cancer susceptibility (Table 2).

### Heterogeneity and publication bias

**Figure 3:**
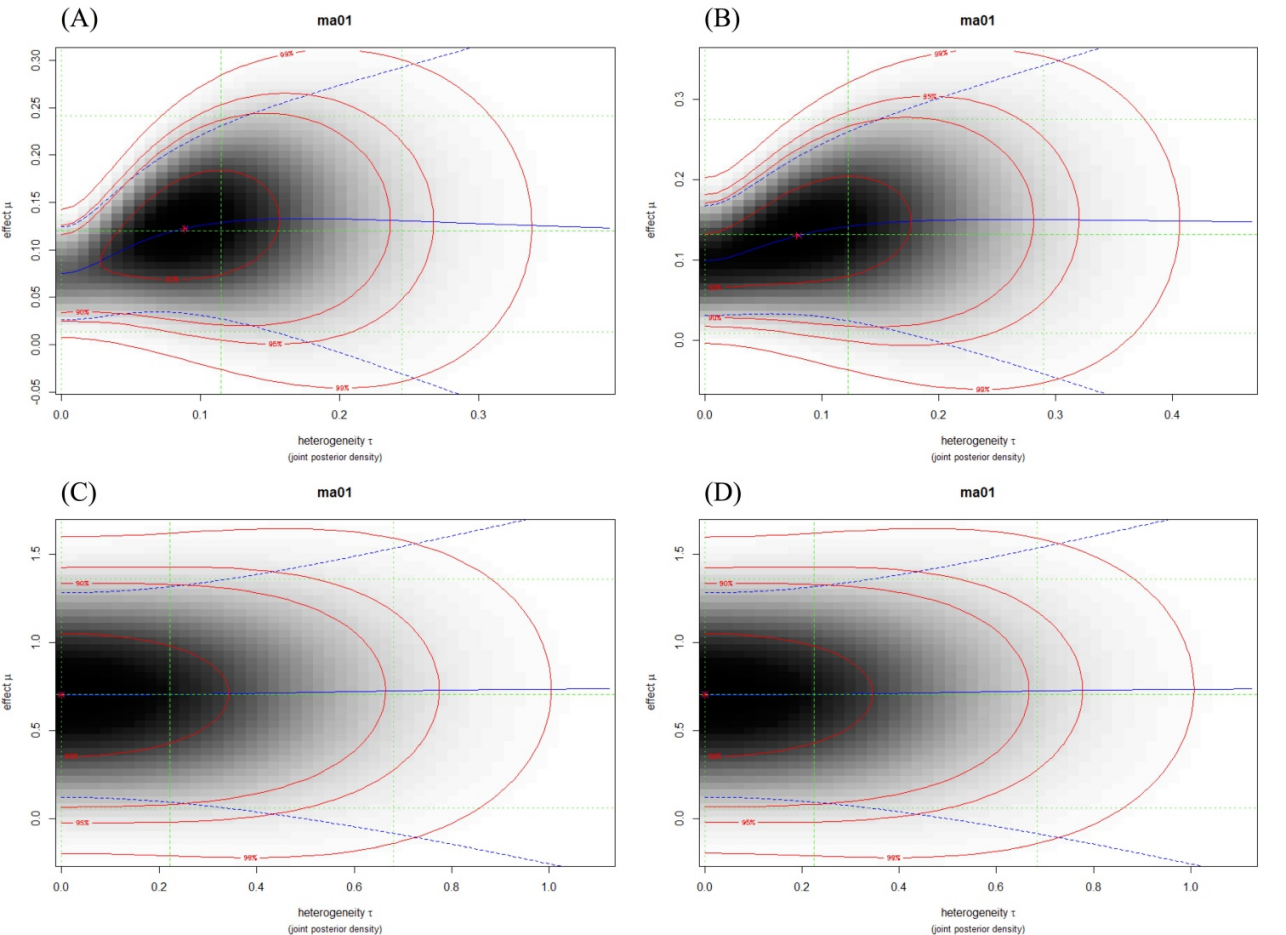
Heterogeneity plots illustrated the joint posterior density of heterogeneity *τ* and effect μ (Log OR), with darker shading corresponding to higher probability density. The red lines indicate (approximate) 2-dimensional credible regions, and the green lines indicate marginal medians and shortest 95% credible intervals for Log OR and *τ*. Blue lines show the conditional mean effect (Log OR) as a function of the heterogeneity *τ* (solid line) along with conditional 95% confidence bounds (dashed lines). (A) rs11077: C vs. A; (B) rs11077: CC+AC vs. AA; (C) rs3803012: GG vs. AA; (D) rs3803012: GG vs. AG+AA

Evaluation of the heterogeneity of the studies was analyzed with the *τ^2^* test. *I*^2^ > 0.50 was considered as high value for heterogeneity. On the basis of heterogeneity plots and *I*^2^ value, in most of the meta-analyses, the total heterogeneity and between studies heterogeneity were not high, for example, rs11077 and rs3803012 (Figure 3, Table 2). However, these results for rs14035 were significantly high (Table 2).

Begg's and Egger's tests were performed to evaluate the potential publication bias. As shown in Table 2, no publication bias was observed in our meta-analysis.

### Functional validation by eQTL analysis

To substantiate the associations between the identified SNPs (XPO5 rs11077 and RAN rs3803012 SNPs) and cancer risk, we performed the eQTL analysis to assess the associations between SNPs and corresponding mRNA expression levels. The eQTL analysis results of lymphoblastoid cell lines from 373 Europeans were visualized through the Geuvadis Data Browser (https://www.ebi.ac.uk/Tools/geuvadis-das), and we found that XPO5 rs11077 was significantly associated with XPO5 mRNA expression levels (*P* = 5.83E-07).

**Figure 4:**
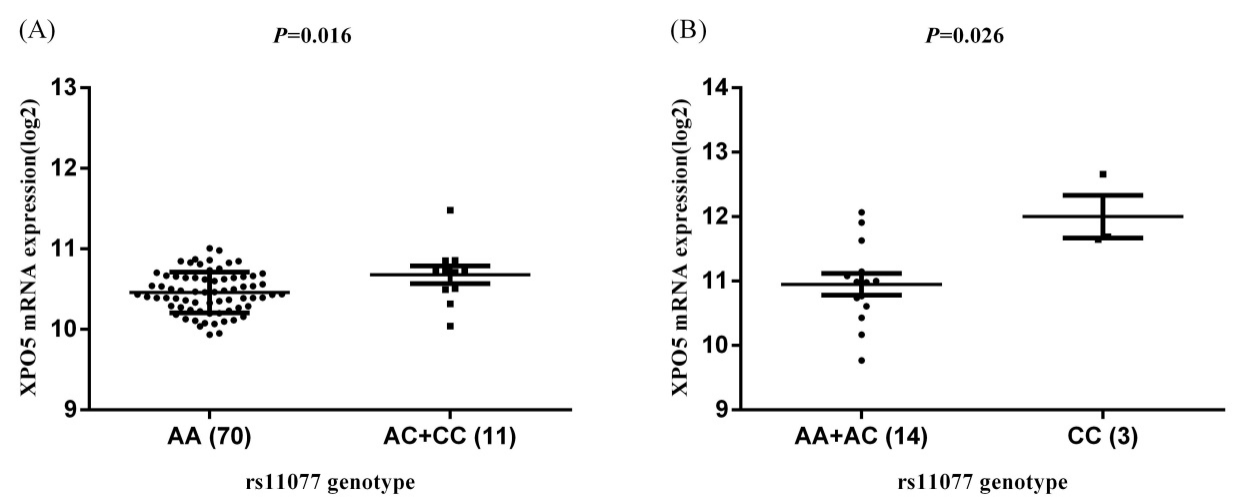
The eQTL analysis of rs11077 with the mRNA expression of XPO5. (A) dominant model in Stranger et al. study 27, *P* = 0.016; (B) recessive model in GEO database (GSE65373), *P* = 0.026

**Figure 5:**
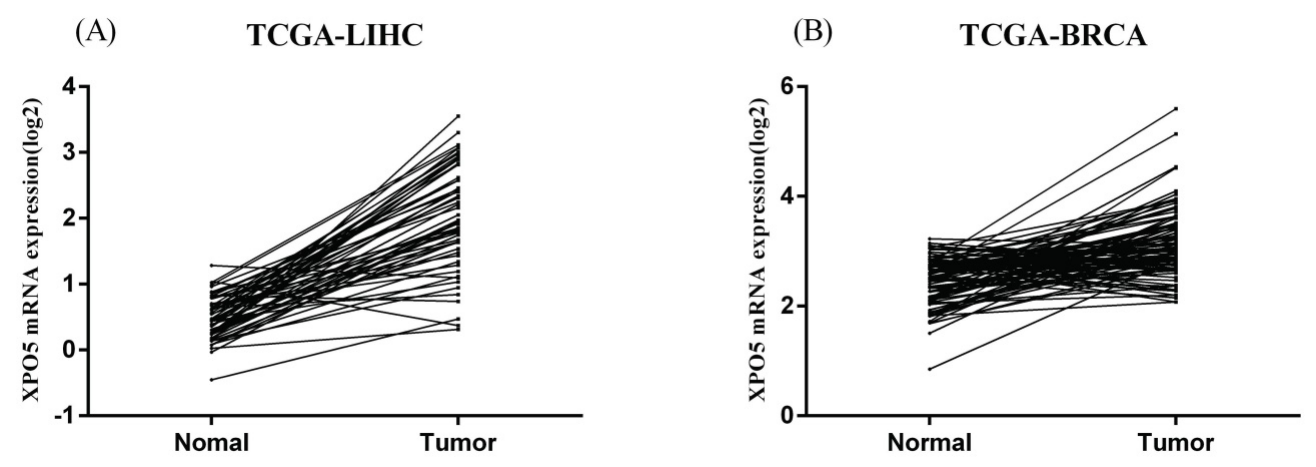
The mRNA expression of XPO5 in the 154 paired cancer tissue samples and normal adjacent tissue samples from the TCGA database. (A) 58 paired hepatocellular carcinoma samples in TCGA-LIHC, *P* = 1.50E-20; (B) 96 paired breast cancer samples in TCGA-BRCA, *P* = 5.27E-11

Similarly, in the HapMap3 East Asian samples (81 Japanese samples) from Stranger et al. 27, rs11077 was significantly associated with the expression of XPO5 gene (*P* = 0.016, Figure 4A), with the risk of C allele predicting higher mRNA levels of XPO5. According to the genotyping and expression data of 17 hepatocellular carcinoma obtained from the GEO database (GSE65373), we also found that rs11077 C allele had a significant association with an increased mRNA expression levels of XPO5 in the recessive model (*P* = 0.026, Figure 4B). However, no significant associations between rs3803012 and RAN mRNA expression levels were found in the above datasets. In addition, we compared mRNA expression levels of XPO5 in 154 paired cancer tissue samples with normal adjacent tissue samples from two TCGA projects (58 paired samples in TCGA-LIHC and 96 paired samples in TCGA-BRCA). We found that XPO5 mRNA expression levels were significantly increased in the tumor tissues compared to the normal tissues (*P* = 1.50E-20 and *P* = 5.27E-11, respectively) (Figure 5).

## Discussion

In the present study, a total of five SNPs in XPO5 and RAN genes were comprehensively reviewed and analyzed to estimate their associations with the risk of overall cancer by Bayesian hierarchical meta-analysis. Of these five SNPs, two (rs2257082, rs3809142) were analyzed for the first time. In contrast to the classical meta-analysis already performed with a fewer number of articles included 13, the Bayesian hierarchical meta-analysis applied here indicated that rs11077 SNP of XPO5 and rs3803012 SNP of RAN might facilitate the carcinogenesis. Nonetheless, we also performed a classical meta-analysis of the current data (results were not mentioned) that demonstrated the association of most of the genetic models in rs11077 SNP and the relevance of the rs3803012 SNP in homozygous and recessive models.

Since the Bayesian hierarchical meta-analysis is much more sensitive and confers more precise estimation compared with classical meta-analysis, it is powerful suggested that rs11077 SNP of XPO5 and rs3803012 SNP of RAN are associated with cancer risk. Furthermore, eQTL analysis demonstrated that rs11077 SNP may influence the mRNA expression levels of XPO5. However, no associations were revealed amongst other studied SNPs in our meta-analysis, therefore future studies with a larger sample size are needed to determine their relationships.

For the classical meta-analysis, when faced with extreme values or small research quantum, the accuracy of the results cannot be guaranteed and the correctness of its conclusions will be questionable 14. However, with the development of Markov Chain Monte Carlo (MCMC) methods, Bayesian hierarchical meta-analysis can avoid these defects and address the actual research question more directly 55-57. Chen et al. 14 compared the difference between fully Bayesian hierarchical meta-analysis and classical meta-analysis, and found that if fixed effect is used to determine the real effect, both types of meta-analysis can be used. When random effect is adopted, if the study quantum is < 20, the Bayesian hierarchical meta-analysis should be the analysis of choice. The number of included articles for each studied SNP was < 20 in our meta-analysis, therefore the Bayesian hierarchical meta-analysis was utilized. From a statistical point of view, the number of included studies was not large enough for a classical meta-analysis, thus the results should be interpreted with caution. In the Bayesian hierarchical meta-analysis, however, the credible interval is slightly wider than that of classical meta-analysis and the results tend to be more consistent 14-15. Hence, the significant result of Bayesian hierarchical meta-analysis is conservative and more reliable in comparison with the classical meta-analysis.

XPO5 is a member of karyopherin β family related to human export receptor CRMI, and is responsible for nuclear export and stabilization to form mature miRNA to produce physiological effects 58-59. As XPO5 is a key factor for the transportation of miRNA from the nucleolus, it has been postulated as a rate-limiting step in the development of miRNAs, so its impairment could lead to pre-miRNA trapping in the nucleolus, influencing the risk of cancer 60-61. Current studies have indicated the role of XPO5 in the development of several sorts of cancers such as hepatocellular carcinoma, thyroid cancer, lung cancer, and so on 62-64. These studies are consistent with the results of the present study in which XPO5 mRNA expression levels were significantly increased in tumor tissues compared to normal tissues in 154 paired cancer tissue samples from the TCGA database. In addition, an increasing number of studies have focused on the correlations of XPO5 polymorphisms with cancer risk. The previous classical meta-analysis of XPO5 gene rs11077 SNP performed by He et al. 13, which included 7 case-control studies, showed no significant correlation with cancer risk. However, our analysis which included 14 case-control studies indicated that the minor C allele of rs11077 SNP significantly increased the risk of cancer and a strong association with cancer risk was also found in the dominant model. This association was further supported by the significant correlation between rs11077 C allele and an increased XPO5 mRNA expression level in the eQTL analysis. These findings suggest that rs11077 was significantly associated with cancer risk possibly by decreasing the mRNA expression levels of XPO5. Located in the 3'-UTR of XPO5, the A to C substitution of rs11077 may affect mRNA stability, alter the expression of XPO5 and, consequently, affect the expression of miRNAs, resulting in an aberrant expression of miRNA target gene at the post-transcriptional level 12,65.

RAN is a key member of the Ras superfamily of GTPases and is essential for translocation of pre-miRNAs from the nucleus to the cytoplasm through the nuclear pore complex in a GTP-dependent manner 66. Studies have revealed that the up-regulation of RAN expression in various malignancies supports its role in cancer development 67-69. No significant association was observed in the RAN gene rs3803012 SNP according to the previous classical meta-analysis 13, which included 5 case-control studies. In contrast, our Bayesian hierarchical meta-analysis which included 6,514 cases and 8,707 healthy subjects for the RAN gene rs3803012 SNP from 7 studies, demonstrated a significant association between rs3803012 SNP (homozygote or recessive model) and overall cancer risk. Our classical meta-analysis (the results of this analysis were not included in this study) also demonstrated a significant increased association risk of RAN gene rs3803012 SNP in cancer. Studies have hypothesized that the RAN rs3803012 G allele might affect the targeting of hsa-miR-199a-3p and result in decreased expression of RAN mRNA in tumor cells, which may affect various miRNA biosynthesis 43. Unfortunately, we failed to obtain a significant eQTL results for SNP rs3803012 because the minor allele frequency (MAF) of rs3803012 was low in the included datasets (MAF ≤ 0.05). Population-specific eQTL analysis are warranted to validate our findings. As for the RAN gene rs14035 and rs3809142 polymorphisms, both types of meta-analysis did not support the significant association with cancer risk. The total heterogeneity as well as between studies heterogeneity was relatively high. Thus, further investigations are required to identify these potential cancer susceptibility loci.

Despite these results, we encountered some limitations during our meta-analysis. Firstly, since we had a limited number of studies, we could not perform a subgroup analysis with respect to the ethnicity, source of control groups (population-based or hospital-based) and cancer type. Heterogeneity among different cancers may cause the real effects to be hidden when pooling all cancer types. Secondly, gene-environmental interactions which may alter cancer risk were not evaluated due to the lack of relevant data across the included studies. Thirdly, studies of XPO5 and RAN SNPs in the cancer predisposition field continue to emerge, which resulted in limited number of the relevant investigations.

In view of all this, Bayesian hierarchical meta-analysis suggests a potential role of the miRNA biogenesis genes XPO5 (rs11077 A/C) and RAN (rs3803012 A/G) SNPs in cancer risk, supplying novel clues to identifying new biomarkers with cancer-forewarning function. Although we used publicly available genotyping and expression data to confirm the biological significance of the variant and suggest that XPO5 rs11077 may be a possible functional susceptibility locus for cancer risk, further high-quality research and functional evaluations are still warranted to validate our findings due to the limitations mentioned above.

## Supplementary Material

Supplementary tables.

## Figures and Tables

**Table 1:** Characteristics of the studies eligible for the meta-analysis

Author	Year	Country	Ethnicity	Cancer type	Source ofcontrols	Genotypingmethod	Case/control	Cases					Controls					P_HWE_	NOS
**XPO5 rs11077**								**AA**	**AC**	**CC**	**A**	**C**	**AA**	**AC**	**CC**	**A**	**C**		
Thakkar *et al.*	2018	India	Asian	Hodgkin lymphoma	PB	TaqMan	101/200	39	41	21	119	83	76	92	32	244	156	0.639	8
Wang *et al.*	2017	China	Asian	Breast cancer	HB	PCR-RFLP	116/120	87	28	1	202	30	103	17	0	223	17	0.401	7
Wen* et al.*	2017	China	Asian	Thyroid cancer	HB	TaqMan	1134/1228	907	210	17	2024	244	1023	194	11	2240	216	0.593	7
Kim* et al.*	2016	China	Asian	Hepatocellular carcinoma	PB	PCR-RFLP	147/209	128	19	0	275	19	170	38	1	378	40	0.465	8
Zhao *et al.*	2015	China	Asian	Colorectal cancer	HB	PCR-LDR	163/142	143	19	1	305	21	123	18	1	264	20	0.705	7
Cho* et al.*	2015	Korea	Asian	Colorectal cancer	HB	PCR-RFLP	408/400	333	74	1	740	76	337	61	2	735	65	0.668	7
Xie* et al.*	2015	China	Asian	Gastric cancer	HB	PCR-LDR	137/142	119	17	1	255	19	123	18	1	264	20	0.705	6
Buas *et al.*	2015	Europe	Caucasian	Esophageal cancer	HB	Illumina	5780/3206	1909	2826	1045	6644	4916	1097	1557	552	3751	2661	0.991	7
Ding* et al.*	2013	China	Asian	Lung cancer	HB	PCR-LDR	112/80	94	18	0	206	18	65	14	1	144	16	0.804	8
Navarro *et al.*	2013	Spain	Caucasian	Hodgkin lymphoma	HB	TaqMan	127/104	25	67	35	117	137	34	46	24	114	94	0.275	7
Sung *et al.*	2011	Korea	Asian	Breast cancer	HB	TaqMan	559/567	473	82	4	1028	90	501	64	2	1066	68	0.977	7
Horikawa* et al.*	2008	USA	Caucasian	Renal cell carcinoma	PB	SNPlex	276/277	86	136	54	308	244	110	129	38	349	205	0.985	8
Yang *et al.*	2008	USA	Caucasian	Bladder cancer	HB	SNPlex	718/726	248	356	114	852	584	241	363	122	845	607	0.457	7
Ye *et al.*	2008	USA	Caucasian	Esophageal cancer	HB	SNPlex	300/295	101	146	53	348	252	118	137	40	373	217	0.981	7
**XPO5 rs2257082**								**GG**	**AG**	**AA**	**G**	**A**	**GG**	**AG**	**AA**	**G**	**A**		
Bermisheva *et al.*	2018	Russia	Caucasian	Breast cancer	NM	PCR	417/361	182	187	48	551	283	172	154	35	498	224	0.951	6
Liang *et al.*	2010	USA	Caucasian	Ovarian cancer	HB	Illumina	339/349	192	126	21	510	168	181	141	27	503	195	0.949	7
Martin-Guerrero* et al.*	2015	Spain	Caucasian	Lymphocytic leukemia	HB	TaqMan	101/346	59	37	5	155	47	202	123	21	527	165	0.694	7
**RAN rs3803012**								**AA**	**AG**	**GG**	**A**	**G**	**AA**	**AG**	**GG**	**A**	**G**		
Wang *et al.*	2016	China	Asian	Hepatocellular carcinoma	HB	TaqMan	312/320	250	56	6	556	68	260	55	5	575	65	0.298	7
Liu *et al.*	2013	China	Asian	Hepatocellular carcinoma	PB	TaqMan	1256/2678	1158	95	3	2411	101	2450	227	1	5127	229	0.066	8
Jiang* et al.*	2013	China	Asian	Breast cancer	PB	TaqMan	870/884	766	92	12	1624	116	772	107	5	1651	117	0.539	8
Zhang* et al.*	2012	China	Asian	Gastric cancer	PB	TaqMan	1654/1844	1517	133	4	3167	141	1674	168	2	3516	172	0.292	8
Li et al.	2012	China	Asian	Hepatocellular carcinoma	PB	PCR-RFLP	560/560	508	52	0	1068	52	512	48	0	1072	48	0.289	8
Chen et al.	2012	China	Asian	Cervical carcinoma	PB	TaqMan	1471/1529	1325	141	5	2791	151	1397	129	3	2923	135	0.990	8
Ma et al.	2012	China	Asian	Head and neck cancer	PB	TaqMan	391/892	344	45	2	733	49	799	91	2	1689	95	0.725	8
**RAN rs14035**								**CC**	**CT**	**TT**	**C**	**T**	**CC**	**CT**	**TT**	**C**	**T**		
Kim et al.	2016	China	Asian	Hepatocellular carcinoma	PB	PCR-RFLP	147/229	98	42	7	238	56	137	69	3	343	75	0.080	8
Meng et al.	2015	China	Asian	Hepatocellular carcinoma	PB	SNPstream	324/343	208	105	11	521	127	229	107	7	565	121	0.172	8
Cho et al.	2015	Korea	Asian	Colorectal cancer	HB	PCR-RFLP	408/400	267	128	13	662	154	233	150	17	616	184	0.240	7
Martin-Guerrero et al.	2015	Spain	Caucasian	Lymphocytic leukemia	HB	TaqMan	99/342	48	41	10	137	61	138	164	40	440	244	0.407	7
Xie et al.	2015	China	Asian	Gastric cancer	HB	PCR-LDR	137/142	86	45	6	217	57	35	71	36	141	143	0.999	6
Buas et al.	2015	Europe	Caucasian	Esophageal cancer	HB	Illumina	5783/3202	2760	2470	553	7990	3576	1525	1370	307	4420	1984	0.978	7
Zhao et al.	2015	China	Asian	Colorectal cancer	HB	PCR-LDR	163/142	113	45	5	271	55	107	33	2	247	37	0.761	7
Roy et al.	2014	India	Asian	Oral cancer	HB	PCR-RFLP	439/438	281	134	24	696	182	301	124	13	726	150	0.958	7
Li et al.	2012	China	Asian	Hepatocellular carcinoma	PB	PCR-RFLP	560/560	376	160	24	912	208	390	160	10	940	180	0.162	8
Kim et al.	2010	Korea	Asian	Lung cancer	HB	MS	93/90	65	23	5	153	33	52	33	5	137	43	0.937	7
Horikawa et al.	2008	USA	Caucasian	Renal cell carcinoma	PB	SNPlex	276/278	143	110	23	396	156	129	125	24	383	173	0.415	8
Ye et al.	2008	USA	Caucasian	Esophageal cancer	HB	SNPlex	304/301	127	139	38	393	215	166	115	20	447	155	0.989	7
**RAN rs3809142**								**CC**	**CT**	**TT**	**C**	**T**	**CC**	**CT**	**TT**	**C**	**T**		
Bermisheva et al.	2018	Russia	Caucasian	Breast cancer	NM	PCR	415/359	313	89	13	715	115	208	130	21	546	172	0.908	6
Jiang et al.	2013	China	Asian	Breast cancer	PB	TaqMan	862/886	602	232	28	1436	288	615	239	32	1469	303	0.149	8

Abbreviations: HB: hospital-based; PB: population-based; HWE: Hardy-Weinberg equilibrium; NOS: Newcastle-Ottawa scale; MS: sequenome MS-based genotyping assay; PCR: polymerase chain reaction; PCR-RFLP: polymerase chain reaction-restriction fragment length polymorphism; PCR-LDR: polymerase chain reaction-ligase detection reaction; NM: not mentioned.

**Table 2:** Bayesian hierarchical meta-analysis of the pooled associations between XPO5 and RAN genes polymorphisms and risk of cancer

SNPs	Variations	Percentage heterogeneity *I^2^*	Association test		Absolute heterogeneitytest *τ^2^* (95% CrI)	Publication bias (Begg's test, *p* value; Egger's test, *p* value)
			Pooled Log OR	95% CrI		
XPO5 rs11077 (A>C)	C vs. A	0.486	**0.120**	0.013, 0.241	0.115 (0.000, 0.245)	0.443, 0.166
	AC vs. AA	0.255	0.110	-0.004, 0.243	0.094 (0.000, 0.254)	0.827, 0.253
	CC vs. AA	0.345	0.216	-0.014, 0.508	0.207 (0.000, 0.470)	0.228, 0.174
	CC+AC vs. AA	0.385	**0.132**	0.009, 0.275	0.122 (0.000, 0.289)	0.827, 0.228
	CC vs. AC+AA	0.193	0.134	-0.042, 0.367	0.126 (0.000, 0.354)	0.324, 0.194
XPO5 rs2257082 (G>A)	A vs. G	0.643	-0.012	-0.405, 0.369	0.181 (0.000, 0.603)	1.000, NA
	AG vs. GG	0.499	-0.003	-0.412, 0.407	0.178 (0.000, 0.616)	1.000, NA
	AA vs. GG	0.384	-0.022	-0.640, 0.557	0.265 (0.000, 0.767)	1.000, NA
	AA+AG vs. GG	0.572	-0.008	-0.434, 0.414	0.197 (0.000, 0.638)	1.000, NA
	AA vs. AG+GG	0.339	-0.025	-0.606, 0.521	0.233 (0.000, 0.727)	1.000, NA
RAN rs3803012 (A>G)	G vs. A	0.187	0.032	-0.104, 0.169	0.070 (0.000, 0.222)	0.707, 0.981
	AG vs. AA	0.232	-0.018	-0.168, 0.136	0.084 (0.000, 0.259)	0.707, 0.919
	GG vs. AA	0.070	**0.707**	0.059, 1.358	0.223 (0.000, 0.681)	0.806, 0.741
	GG+AG vs. AA	0.208	0.007	-0.137, 0.155	0.077 (0.000, 0.243)	0.707, 0.983
	GG vs. AG+AA	0.071	**0.708**	0.059, 1.359	0.224 (0.000, 0.684)	0.806, 0.774
RAN rs14035 (C>T)	T vs. C	0.933	-0.068	-0.354, 0.213	0.435 (0.251, 0.683)	0.115, 0.676
	CT vs. CC	0.861	-0.125	-0.396, 0.128	0.373 (0.166, 0.637)	0.193, 0.326
	TT vs. CC	0.859	0.082	-0.457, 0.623	0.790 (0.449, 1.195)	0.150, 0.631
	TT+CT vs. CC	0.919	-0.122	-0.445, 0.190	0.482 (0.266, 0.763)	0.244, 0.465
	TT vs. CT+CC	0.791	0.142	-0.301, 0.593	0.606 (0.267, 1.005)	0.193, 0.464
RAN rs3809142 (C>T)	T vs. C	0.941	-0.333	-1.169, 0.487	0.453 (0.117, 0.998)	1.000, NA
	CT vs. CC	0.927	-0.373	-1.265, 0.497	0.494 (0.137, 1.043)	1.000, NA
	TT vs. CC	0.566	-0.427	-1.296, 0.382	0.363 (0.000, 0.933)	1.000, NA
	TT+CT vs. CC	0.934	-0.388	-1.280, 0.486	0.498 (0.144, 1.043)	1.000, NA
	TT vs. CT+CC	0.470	-0.326	-1.117, 0.423	0.297 (0.000, 0.868)	1.000, NA

Abbreviations: Marginal posterior summary, bold pooled Log OR indicated as statistically significant at 0.05 level. *I*^2^: relative heterogeneity; CrI: credible interval; OR: odds ratio; SNP: single nucleotide polymorphism; NA: not available.
